# CYP2C19 loss-of-function alleles predicts clinical outcomes in East Asian patients with acute myocardial infarction undergoing percutaneous coronary intervention and stenting receiving clopidogrel

**DOI:** 10.3389/fcvm.2022.994184

**Published:** 2022-08-22

**Authors:** Yu-Wei Chen, Yi-Ju Liao, Wei-Chun Chang, Tzu-Hung Hsiao, Ching-Heng Lin, Chiann-Yi Hsu, Tsun-Jui Liu, Wen-Lieng Lee, Yi-Ming Chen

**Affiliations:** ^1^Cardiovascular Center, Taichung Veterans General Hospital, Taichung, Taiwan; ^2^Institute of Clinical Medicine, National Yang Ming Chiao Tung University, Taipei, Taiwan; ^3^Department of Post-Baccalaureate Medicine, College of Medicine, National Chung Hsing University, Taichung, Taiwan; ^4^Department of Pharmacy, Taichung Veterans General Hospital, Taichung, Taiwan; ^5^Department of Cardiology, Feng Yuan Hospital, Ministry of Health and Welfare, Taichung, Taiwan; ^6^Department of Life Science, Tunghai University, Taichung, Taiwan; ^7^Department of Medical Research, Taichung Veterans General Hospital, Taichung, Taiwan; ^8^Department of Public Health, Fu Jen Catholic University, New Taipei City, Taiwan; ^9^Institute of Genomics and Bioinformatics, National Chung Hsing University, Taichung, Taiwan; ^10^Institute of Public Health and Community Medicine Research Center, National Yang Ming Chiao Tung University, Taipei, Taiwan; ^11^Department of Industrial Engineering and Enterprise Information, Tunghai University, Taichung, Taiwan; ^12^Biostatistics Task Force of Taichung Veterans General Hospital, Taichung, Taiwan; ^13^School of Medicine, College of Medicine, National Yang Ming Chiao Tung University, Taipei, Taiwan; ^14^Division of Allergy, Immunology and Rheumatology, Department of Internal Medicine, Taichung Veterans General Hospital, Taichung, Taiwan; ^15^Ph.D. Program in Translational Medicine, Rong Hsing Research Center for Translational Medicine, National Chung Hsing University, Taichung, Taiwan

**Keywords:** clopidogrel, coronary artery disease, CYP2C19, dual antiplatelet therapy, P2Y12 inhibitors

## Abstract

**Background:**

CYP2C19 loss-of-function (LOF) alleles reduce the effectiveness of clopidogrel and are associated with high rates of clinical events in patients undergoing percutaneous coronary intervention (PCI) and stenting in Northeast Asians. However, the prevalence and influence of CYP2C19 LOF alleles in Southeast Asians remain unclear.

**Objectives:**

This study aims to retrospectively investigate the prevalence of CYP2C19 LOF alleles and clinical outcomes in East Asian patients taking clopidogrel and undergoing PCI.

**Methods:**

Between June 2019 and June 2020, volunteer participants in a single medical center were consecutively selected. The genetic data of CYP2C19 were derived from the Taiwan Precision Medicine Initiative (TPMI). Patients receiving clopidogrel while undergoing PCI with stenting were retrospectively analyzed.

**Results:**

A total of 999 patients (62.4 ± 11.1 years old, 83.7% men) were enrolled; 39.3% without the CYP2C19 LOF allele (normal metabolizers + rapid metabolizers, NM + RM); 44.9% with one LOF allele (intermediate metabolizers, IM); 15.7% with two LOF alleles (poor metabolizers, PM). The incidence of stroke was higher in the PM subgroup compared to the NM + RM subgroup or IM subgroup in patients presenting with acute myocardial infarction (AMI). The 1-year major adverse cardiac and cerebrovascular events (MACCE)-free survival rates in all participants were similar among the three groups. However, in the AMI group, the 1-year MACCE-free survival rates were significantly lower in the PM subgroup compared to the NM + RM subgroup or IM subgroup.

**Conclusion:**

In East Asians presenting with AMI, CYP2C19 PM was associated with deleterious cardiovascular outcomes and stroke. Our results reinforce the crucial role of preemptive CYP2C19 genotyping in East Asian AMI patients receiving clopidogrel treatment.

## Introduction

For patients with coronary artery disease who underwent percutaneous coronary intervention (PCI) and stenting, dual antiplatelet therapy (DAPT) is mandatory to prevent stent thrombosis and in-stent restenosis. Clopidogrel as an adjunct to aspirin therapy has been shown to reduce clinical cardiovascular ischemic events in patients with coronary stenting (1, 2). DAPT with aspirin and clopidogrel is used exclusively in patients with chronic coronary syndrome and in selected patients with acute coronary syndrome (3). However, the response to clopidogrel is variable among patients, and individuals with resistance to clopidogrel are at an increased risk of recurrent atherothrombotic events (4).

Clopidogrel is a prodrug of the thienopyridine class, which is sequentially converted to its active metabolite in the liver by two cytochrome P450 (CYP) isoenzymes that are encoded by polymorphic genes (5). Among these genes, carriers with at least one reduced function of the CYP2C19 alleles are associated with a consistent attenuation of pharmacokinetic and pharmacodynamic responses to clopidogrel (6). Furthermore, these patients have a higher rate of major adverse cardiovascular events, including stent thrombosis (6, 7).

In East Asian patients who are treated with clopidogrel after PCI, the response to clopidogrel is variable and controversial. The prevalence of CYP2C19 loss-of-function (LOF) alleles is substantially higher in East Asians, compared to patients from other geographic regions (8); thus, more East Asian patients have high on-treatment platelet reactivity. In contrast, East Asian patients have lower stent thrombosis and ischemic events than patients in other geographical regions. Some experts argue that the “therapeutic window” in East Asian patients could differ from that in patients of other ethnic groups (5).

This study was conducted with the aim to evaluate the CYP2C19 genotypes and the ischemic outcomes of post-PCI clopidogrel treatment in East Asian patients.

## Materials and methods

### Study population

Patients who visited outpatient clinics at Taichung Veterans General Hospital (TCVGH), a tertiary teaching hospital in central Taiwan, were invited to participate in the Taiwan Precision Medicine Initiative (TPMI), a nationwide genetic project led by Academia Sinica and partner hospitals. Blood samples from each participant in the TPMI were collected, and DNA was extracted and genotyped. The genetic profiles were linked to their electronic health records in TCVGH, including medical history, biochemical reports, and coronary artery angiography reports. The detailed process of data collection has been reported previously (9).

Between June 2019 and June 2020, TPMI participants from TCVGH who used clopidogrel for at least 4 weeks from the outpatient clinic and underwent PCI for coronary artery lesions in the setting of stable coronary artery disease or acute coronary syndrome were enrolled in this study. This study was conducted in an all-comer design and patients with an undefined CYP2C19 phenotype were excluded. The study protocol was approved by the Institutional Review Board of TCVGH (CE20316A). Informed consents were obtained in accordance with the principles defined in the Declaration of Helsinki.

### Study endpoints

The endpoints of this study were a composite of major adverse cardiac and cerebrovascular events (MACCE) at 1 year, including non-fatal myocardial infarction, target vessel revascularization, stent thrombosis, or stroke.

### Genotyping and phenotyping of CYP2C19

The single-nucleotide polymorphism (SNP) array TWBv2 (Thermo Fisher Scientific, Inc., Santa Clara, CA, United States), which contains ∼415,000 markers for whole genome sequencing (WGS) and imputation, was designed for both genome-wide association studies (GWAS) and the test of known risk alleles. The high coverage, WGS data and genome-wide SNP data from large-scale Han Chinese ancestry using these custom arrays has been prescribed (10). We genotyped 32,728 participants in TPMI using the TWBv2 array; thirty of them who had an undefined CYP2C19 phenotype were excluded. The remaining 32,698 participants were classified according to CYP2C19 genotypes, including three variants known to have decreased CYP2C19 function [rs4244285 (*2), rs4986893 (*3) and rs72552267 (*6)], along with one variant with increased function [rs12248560 (*17)]. Subsequently, the phenotypes were defined according to the guidelines of the Clinical Pharmacogenetics Implementation Consortium (CPIC) (11, 12). Participants with one increased function and one normal function allele (*1/*17) were classified as rapid metabolizers (RM). Those who carried two wild-type alleles (*1/*1) were classified as normal metabolizers (NM). Those with one LOF allele and one normal function allele (*1/*2, *1/*3, *1/*6) were classified as intermediate metabolizers (IM), while those with two LOF alleles (*2/*2, *2/*3, *3/*3, *2/*6 *3/*6, *6/*6) were classified as poor metabolizers (PM).

### Baseline comorbidities and laboratory data collection

We extracted comorbidity data according to the international statistical classification of diseases and related health problems (ICD) 10th revision (ICD-10) and ICD-9 diagnostic codes from the electronic health records of TCVGH. Comorbidities of coronary artery disease (I20-I25/410-414), heart failure (I50.9/428), hypertension (I10-I13.2/401-404.93), diabetes mellitus (E08-E13/250), hyperlipidemia (E78.0-E78.5/414.00-414.05), stroke (I63/433.X1,434.X1), peripheral artery disease (I73/443.9), and chronic kidney disease (N18/585) were judged if the diagnostic codes (ICD-10 or ICD-9) presented at least twice in the medical records in the outpatient departments before index PCI. If the patient was hospitalized, the comorbidities were judged if the diagnostic codes presented once in the medical records during hospitalization before index PCI. The patients’ hemoglobin level, lipid profile, HbA1C level, and serum creatinine level as well as peak values of cardiac enzymes were collected from the electronic health records.

### Statistical analysis

Continuous variables were expressed as mean ± standard deviation if they were normally distributed in the Kolmogorov–Smirnov test. Categorical variables were expressed as numbers and percentages. The intergroup differences in continuous variables were analyzed using the non-parametric Mann–Whitney *U*-test or the Kruskal–Wallis test. Differences in categorical variables were analyzed using the chi-square test or Fisher’s exact test. Multivariate Cox regression analysis was performed to identify independent predictors of MACCE at 1 year with the adjustment for variables with *p*-values < 0.05 in the univariate analysis. Two-tailed *p*-values < 0.05 were considered statistically significant. Kaplan-Meier survival analysis was used to compare MACCE-free survival among participants with different CYP2C19 phenotypes. All statistical analyzes were performed using IBM SPSS statistical software for Windows, version 22.0 (IBM corp., Armonk, NY, United States).

## Results

### Genotypes and phenotypes of CYP2C19

**FIGURE 1:**
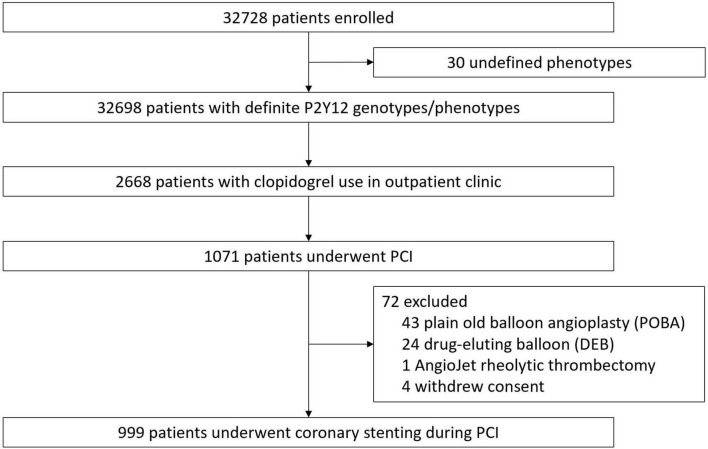
Flowchart of the enrollment process in the study.

The summarized study flow is illustrated in Figure 1. During the study period, a total of 32,698 participants were successfully genotyped for the CYP2C19 gene. Among them, 1,071 clopidogrel users underwent PCI at our institute. Those patients who were treated without stenting were excluded, including 43 patients undergoing plain old balloon angioplasty (POBA), 24 patients undergoing drug-eluting balloon, as well as one patient undergoing AngioJet Rheolytic Thrombectomy (Boston Scientific, Marlborough, MA, United States) as a bailout procedure for a large thrombus burden. During follow-up, four patients requested to withdraw from the study. A total of 999 patients (62.4 ± 11.1 years old, 83.7% men) were enrolled. The details of the genotypes and phenotypes are presented in Table 1. Patients without the CYP2C19 LOF allele (normal metabolizers + rapid metabolizers, NM + RM) accounted for 39.3%, those with one LOF allele (intermediate metabolizers, IM) accounted for 44.9% and those with two LOF alleles (poor metabolizers, PM) accounted for 15.7% of the study population.

**TABLE 1:** Genotypes and phenotypes of the participants.

Phenotype	Genotype	Number (%)
**Rapid metabolizer**		**6 (0.6%)**
	*1/*17	6 (100%)
**Normal metabolizer**		**387 (38.7%)**
	*1/*1	387 (100%)
**Intermediate metabolizer**		**449 (44.9%)**
	*1/*2	397 (88.4%)
	*1/*3	48 (10.7%)
	*2/*17	4 (0.9%)
**Poor metabolizer**		**157 (15.7%)**
	*2/*2	114 (72.6%)
	*2/*3	38 (24.2%)
	*2/*6	2 (1.3%)
	*3/*3	1 (0.6%)
	Novel*	2 (1.3%)

*Novel genotype: rs4244285 (*2) homozygous and rs72552267 (*6) heterozygous. The bold values means the number (%) of each phenotypes.

### Baseline demographic characteristics

The baseline demographic characteristics of the participants are shown in Table 2. In this study, 84.5% of the patients had hypertension, 59.1% had diabetes mellitus, and 21.8% had heart failure. Nearly one third of the participants underwent PCI in the setting of acute coronary syndrome (12.6% STEMI, 10.1% non-STEMI and 12.2% unstable angina). The baseline comorbidities and presentations were similar among the three groups

**TABLE 2:** Baseline demographics of the participants.

Variables	Total (*n* = 999)	NM + RM (*n* = 393)	IM (*n* = 449)	PM (*n* = 157)	*P-value*
Age, years, mean ± *SD*	62.4 ± 11.1	62.8 ± 11.1	61.8 ± 11.1	63.0 ± 11.4	0.184
Male, *n* (%)	836 (83.7%)	330 (84.0%)	380 (84.6%)	126 (80.3%)	0.434
BMI, kg/m^2^, mean ± *SD*	26.1 ± 3.6	26.2 ± 3.4	26.0 ± 3.7	26.5 ± 3.9	0.625
Smoker, *n* (%)	507 (50.8%)	193 (49.1%)	228 (50.8%)	86 (54.8%)	0.486
Hypertension, *n* (%)	844 (84.5%)	335 (85.2%)	371 (82.6%)	138 (87.9%)	0.253
Diabetes mellitus, *n* (%)	590 (59.1%)	241 (61.3%)	257 (57.2%)	92 (58.6%)	0.481
Hyperlipidemia, *n* (%)	844 (84.5%)	335 (85.2%)	377 (84.0%)	132 (84.1%)	0.867
Heart failure, *n* (%)	218 (21.8%)	79 (20.1%)	93 (20.7%)	46 (29.3%)	0.046*
PAD, *n* (%)	44 (4.4%)	19 (4.8%)	17 (3.8%)	8 (5.1%)	0.684
CKD, *n* (%)	115 (11.5%)	45 (11.5%)	52 (11.6%)	18 (10.5%)	0.998
**Presentation**					0.310
STEMI, *n* (%)	126 (12.6%)	49 (12.5%)	57 (12.7%)	20 (12.7%)	
Non-STEMI, *n* (%)	101 (10.1%)	25 (6.4%)	56 (12.5%)	20 (12.7%)	
Unstable angina, *n* (%)	122 (12.2%)	47 (12.0%)	57 (12.7%)	18 (11.5%)	
Stable angina, *n* (%)	565 (56.6%)	239 (60.8%)	239 (53.2%)	87 (55.4%)	
Ischemic CM, *n* (%)	77 (7.7%)	30 (7.6%)	36 (8.0%)	11 (7.0%)	
Other, *n* (%)	8 (0.8%)	3 (0.8%)	4 (0.9%)	1 (0.6%)	
Concomitant OAC, *n* (%)	74 (7.4%)	28 (7.1%)	29 (6.5%)	17 (10.8%)	0.191

*P-values of the overall comparisons among the three groups. An unplanned post hoc pairwise multiple comparison showed no intergroup difference. BMI, body mass index; CKD, chronic kidney disease; CM, cardiomyopathy; IM, intermediate metabolizer; NM, normal metabolizer; non-STEMI, non-ST-elevation myocardial infarction; OAC, oral anticoagulant; PAD, peripheral arterial disease; PM, poor metabolizer; RM, rapid metabolizer; STEMI, ST-elevation myocardial infarction.

### Baseline laboratory and angiographic characteristics

The baseline laboratory and angiographic characteristics of the participants are shown in Table 3. In this study, half of the participants had multivessel disease (52.1%), 74.5% of the patients had LAD involvement, and 70.4% of the patients were treated with second-generation DES. In general, all three groups had similar baseline hemoglobin, lipid profile, HbA1C, renal function, peak values of cardiac enzymes, as well as angiographic characteristics.

**TABLE 3:** Baseline laboratory and angiographic characteristics.

Variables	Total (*n* = 999)	NM + RM (*n* = 393)	IM (*n* = 449)	PM (*n* = 157)	*P-value*
Hb (g/dL), mean ± *SD*	13.5 ± 2.0	13.5 ± 2.0	13.5 ± 2.0	13.3 ± 1.9	0.464
HbA1C (%), mean ± *SD*	6.6 ± 1.4	6.5 ± 1.4	6.5 ± 1.4	6.7 ± 1.4	0.226
Cholesterol (mg/dL), mean ± *SD*	166.0 ± 41.2	167.5 ± 44.8	163.3 ± 37.6	170.2 ± 41.7	0.215
LDL (mg/dL), mean ± *SD*	99.4 ± 35.5	100.3 ± 37.8	98.0 ± 34.4	100.9 ± 33.0	0.555
HDL (mg/dL), mean ± *SD*	42.5 ± 11.0	42.6 ± 11.1	42.1 ± 10.5	43.7 ± 12.0	0.570
TG (mg/dL), mean ± *SD*	149.2 ± 107.4	145.9 ± 100.1	150.2 ± 110.0	154.5 ± 117.6	0.969
eGFR (mL/min/1.73m^2^), mean ± *SD*	74.7 ± 31.0	74.1 ± 28.2	76.2 ± 32.4	72.2 ± 33.4	0.276
CK (U/L), mean ± *SD*	493.1 ± 1037.8	483.3 ± 936.9	467.7 ± 966.6	584.0 ± 1385.7	0.878
CK-MB (U/L), mean ± *SD*	21.3 ± 46.7	22.8 ± 44.4	19.5 ± 41.4	23.1 ± 63.0	0.720
Troponin T (ng/mL), mean ± *SD*	217.0 ± 960.9	362.5 ± 1390.9	155.6 ± 665.3	79.9 ± 215.4	0.801
Troponin I (ng/mL), mean ± *SD*	9.0 ± 23.9	9.2 ± 22.1	8.2 ± 23.4	11.3 ± 29.6	0.707
**CAD numbers, *n* (%)**					0.180
One	478 (47.9%)	186 (47.3%)	208 (46.3%)	84 (53.5%)	
Two	331 (33.1%)	130 (33.1%)	147 (32.7%)	54 (34.4%)	
Three	190 (19.0%)	77 (19.6%)	94 (20.9%)	19 (12.1%)	
**Artery involved, *n* (%)**					
LM	63 (6.3%)	24 (6.1%)	29 (6.5%)	10 (6.4%)	0.978
LAD	744 (74.5%)	292 (74.4%)	346 (77.1%)	106 (67.5%)	0.061
RCA	501 (50.2%)	206 (52.4%)	219 (48.8%)	76 (48.4%)	0.512
LCX	452 (45.3%)	171 (43.5%)	217 (48.3%)	64 (40.8%)	0.176
DEB	34 (3.4%)	9 (2.3%)	21 (4.7%)	4 (2.6%)	0.132
BMS	255 (25.5%)	88 (22.4%)	122 (27.2%)	45 (28.7%)	0.175
BVS	16 (1.6%)	4 (1.0%)	10 (2.2%)	2 (1.3%)	0.355
DES-1st generation	66 (6.6%)	31 (7.9%)	23 (5.1%)	12 (7.6%)	0.232
DES-2nd generation	703 (70.4%)	287 (73.0%)	311 (69.3%)	105 (66.9%)	0.285

BMS, bare metal stent; BVS, bioresorbable vascular scaffold; CAD, coronary artery disease; CK, creatine kinase; CK-MB, creatine kinase-MB isoenzyme; DEB, drug-eluting balloon; DES, drug-eluting stent; eGFR, estimated glomerular filtration rate; IM, intermediate metabolizer; HbA1C, glycated hemoglobin; HDL, high-density lipoprotein; LAD, left anterior descending artery; LCX, left circumflex artery; LDL, low-density lipoprotein; LM, left main coronary artery; NM, normal metabolizer; PM, poor metabolizer; RCA, right coronary artery; RM, rapid metabolizer; TG, triglyceride.

### Clinical outcomes of the whole cohort and of the acute myocardial infarction group

**FIGURE 2:**
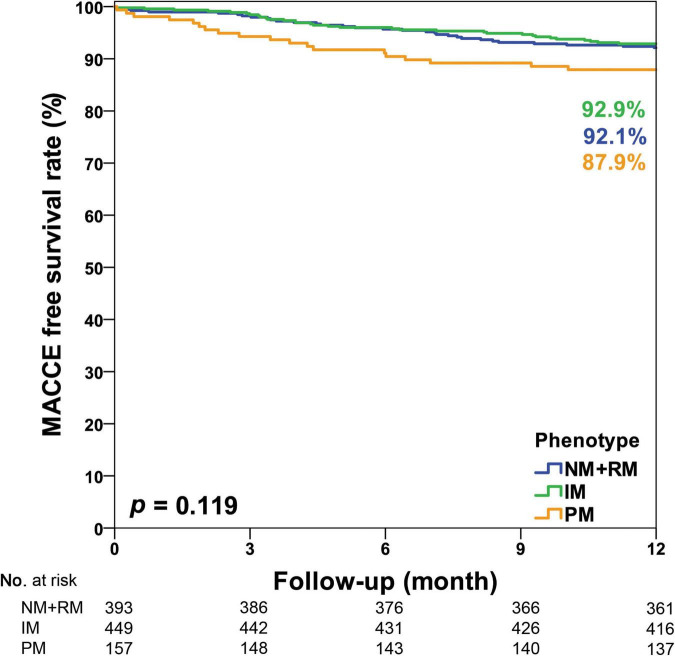
Kaplan–Meier curve of the cumulative incidence of major adverse cardiovascular and cerebrovascular events (MACCE) at the 1-year follow-up stratified based on the CYP2C19 phenotype of all study participants. IM, intermediate metabolizer; NM, normal metabolizer; PM, poor metabolizer; RM, rapid metabolizer.

**FIGURE 3:**
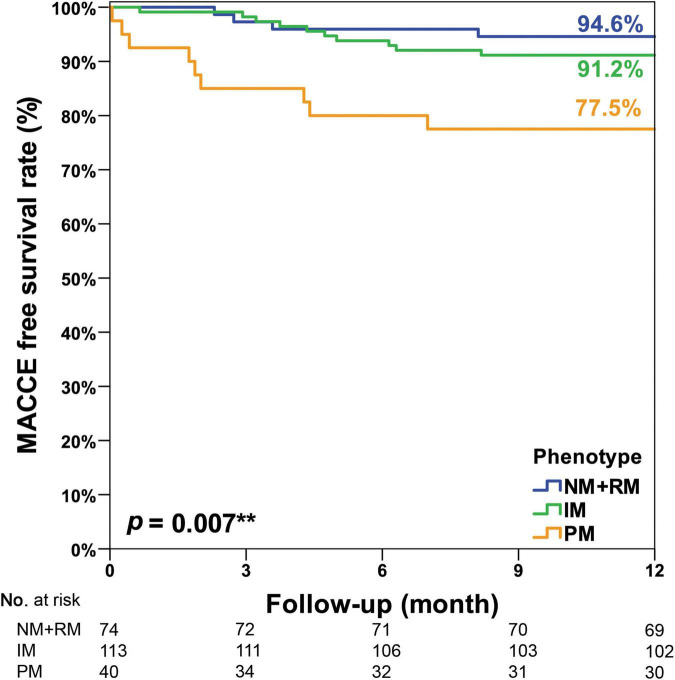
Kaplan–Meier curve of the cumulative incidence of major adverse cardiovascular and cerebrovascular events (MACCE) at the 1-year follow-up stratified based on the CYP2C19 phenotype of the AMI subgroup. An unplanned *post hoc* pairwise multiple comparison showed that there were significantly less MACCE in the NM + RM group than in the PM group (*p* = 0.005). There were significantly less MACCE in the IM group than in the PM group (*p* = 0.016). However, there was no intergroup difference between the NM + RM group and the IM group (*p* = 0.393). IM, intermediate metabolizer; NM, normal metabolizer; PM, poor metabolizer; RM, rapid metabolizer. **Means statistically significant.

In this study, CYP2C19 phenotypes were not associated with 1-year MACCE-free survival among all participants (NM + RM: 92.9% vs. IM: 92.1% vs. PM: 87.9%, *p* = 0.119, Figure 2). In the acute myocardial infarction (AMI) subgroup, we observed a significant association of CYP2C19 phenotypes and 1-year MACCE-free survival (NM + RM: 94.6% vs. IM: 91.2% vs. PM: 77.5%, *p* = 0.007, Figure 3). The incidence of stroke was higher in the PM subgroup compared to the NM + RM subgroup or the IM subgroup (PM group 5.0% vs. NM + RM group 0% vs. IM group 0%, *p* = 0.009). No intergroup differences were found with regard to non-fatal myocardial infarction, target vessel revascularization, stent thrombosis, and coronary artery bypass graft (Table 4).

**TABLE 4:** Clinical outcomes of all participants and of the AMI subgroup.

All participants (*n* = 999)					
**Variables**	**Total (*n* = 999)**	**NM + RM (*n* = 393)**	**IM (*n* = 449)**	**PM (*n* = 157)**	***P-value***
MACCE	82 (8.2%)	31 (7.9%)	32 (7.1%)	19 (12.1%)	0.142
Non-fatal MI*	3 (0.3%)	1 (0.3%)	0	2 (1.3%)	0.042
TVR	68 (6.8%)	25 (6.4%)	30 (6.7%)	13 (8.3%)	0.715
Sent thrombosis	2 (0.2%)	1 (0.3%)	0	1 (0.6%)	0.293
CABG	4 (0.4%)	1 (0.3%)	1 (0.2%)	2 (1.3%)	0.168
Stroke	10 (1.0%)	6 (1.5%)	1 (0.2%)	3 (1.9%)	0.076
**AMI subgroup (*n* = 227)**					
**Variables**	**Total (*n* = 227)**	**NM + RM (*n* = 74)**	**IM (*n* = 113)**	**PM (*n* = 40)**	***P-value***
MACCE^†^	23 (10.1%)	4 (5.4%)	10 (8.9%)	9 (22.5%)	0.013
Non-fatal MI	1 (0.4%)	1 (1.4%)	0	0	0.354
TVR	20 (8.8%)	4 (5.4%)	9 (8.0%)	7 (17.5%)	0.085
Sent thrombosis	1 (0.4%)	0	0	1 (2.5%)	0.096
CABG	3 (1.3%)	1 (1.4%)	1 (0.9%)	1 (2.5%)	0.744
Stroke^‡^	2 (0.9%)	0	0	2 (5.0%)	0.009

Data are presented as number with percentage. *P-values of the overall comparisons among the three groups. An unplanned post hoc, pairwise multiple comparison showed no intergroup difference. ^†^P-values of the overall comparisons among the three groups. An unplanned post hoc pairwise multiple comparison showed that there were significantly less major adverse cardiovascular and cerebrovascular events in the NM + RM group than in the PM group (p = 0.033). ^‡^P-values of the overall comparisons among the three groups. An unplanned post hoc, pairwise multiple comparison showed no intergroup difference. CABG, coronary artery bypass graft; IM, intermediate metabolizer; MACCE, major adverse cardiovascular and cerebrovascular events; MI, myocardial infarction; NM, normal metabolizer; PM, poor metabolizer; RM, rapid metabolizer; TVR, target vessel revascularization.

### Multivariate logistic regression analyses for the 1-year major adverse cardiac and cerebrovascular events

Multivariate analysis identified independent predictors for MACCE at the 1-year follow-up as follows: left main lesion (HR: 2.69; 95% CI: 1.42–5.10; *p* = 0.002), drug-eluting balloon (HR: 2.61; 95% CI: 1.13–6.00; *p* = 0.024), and bare metal stent (HR: 2.85; 95% CI: 1.84–4.42; *p* < 0.001) (Table 5). In the AMI subgroup, CYP2C19 PM (compared to NM + RM; HR: 4.01; 95% CI: 1.18–13.64; *p* = 0.026) were associated with a markedly increased risk of MACCE at 1 year. Triple-vessel disease (compared to single-vessel disease; HR: 3.10; 95% CI: 1.04–9.28; *p* = 0.042) and bare-metal stent (HR: 2.55; 95% CI: 1.08–6.04; *p* = 0.033) were other predictors of the 1-year MACCE (Table 5).

**TABLE 5:** Predictors of major adverse cardiovascular and cerebrovascular events (MACCE) during 1-year follow-up in all participants (*n* = 999) and in the AMI subgroup.

All participants (*n* = 999)				
**Predictors**	**Unadjusted HR (95% CI)**	***p*-value**	**Adjusted HR (95% CI)**	***P-value***
**Phenotype**				
NM + RM	Reference			
IM	0.90 (0.55–1.47)	0.674		
PM	1.60 (0.90–2.83)	0.108		
**CAD number**				
One	Reference			
Two	1.59 (0.94–2.68)	0.083		
Three	2.54 (1.48–4.34)	0.001		
**LM**	2.38 (1.26–4.49)	0.007	2.69 (1.42–5.10)	0.002
**DEB**	2.37 (1.03–5.45)	0.041	2.61 (1.13–6.00)	0.024
**BMS**	2.66 (1.72–4.10)	<0.001	2.85 (1.84–4.42)	<0.001
**AMI subgroup (*n* = 227)**				
**Predictors**	**Unadjusted HR (95% CI)**	***p*-value**	**Adjusted HR (95% CI)**	***P-value***
**Phenotype**				
NM + RM	Reference		Reference	
IM	1.65 (0.52–5.26)	0.398	1.44 (0.44–4.67)	0.543
PM	4.75 (1.46–15.44)	0.010	4.01 (1.18–13.64)	0.026
**CAD number**				
One	Reference		Reference	
Two	3.41 (1.24–9.39)	0.017	2.60 (0.93–7.32)	0.069
Three	3.10 (1.04–9.23)	0.042	3.10 (1.04–9.28)	0.042
**BMS**	2.88 (1.22–6.80)	0.016	2.55 (1.08–6.04)	0.033

BMS, bare metal stent; CAD, coronary artery disease; DEB, drug-eluting balloon; IM, intermediate metabolizer; LM, left main artery; NM, normal metabolizer; PM, poor metabolizer; RM, rapid metabolizer.

## Discussion

Our retrospective study demonstrated several important findings regarding the prevalence and ischemic outcomes among patients receiving clopidogrel after PCI in East Asians: (1) The prevalence of CYP2C19 LOF was high. Approximately 45% of the participants had one LOF allele (IM) and 16% of the participants had two LOF alleles (PM); (2) The 1-year MACCE-free survival rates were similar in the PM group compared to the NM + RM group or IM group; (3) In patients with AMI, the 1-year MACCE rates as well as the incidence of stroke, were significantly higher in the PM subgroup compared to the NM + RM subgroup or IM subgroup; (4) CYP2C19 poor metabolizer was a strong predictor for MACCE in patients presenting with AMI. In summary, our results supported the hypothesis that carrying the CYP2C19 LOF alleles exhibits a significant association with adverse cardiovascular outcomes in East Asians using clopidogrel after PCI in AMI.

The prevalence of CYP2C19 LOF alleles (IM and PM) accounted for one third of the cohorts in Italy (13) and nearly 30% of those in the United States (8). Patients with two LOF alleles (PM) only represented less than 5% of the Western population (8, 13). However, Asian populations had a substantially higher prevalence of carriers of CYP2C19 LOF, representing 60–65% of all patients with 10–15% harboring two LOF alleles (8). Nearly 60% of the patients in our cohort had LOF alleles (IM and PM), and 16% harboring two LOF alleles (PM), in line with Asian and Taiwanese populations (9, 10, 14, 15). Therefore, the clinical outcomes in Asians might be different from those of the western population due to the high prevalence of CYP2C19 LOF.

A meta-analysis showed that CYP2C19 LOF carriers had a higher association with adverse cardiovascular outcomes in Asian populations using clopidogrel after PCI (16). Analysis of stent thrombosis outcomes supported differences in the effect size of CYP2C19 LOF allele carriers between Asian (RR 4.88) and white populations (RR of 1.73) (16). In our study, the 1-year MACCE-free survival rates in patients with AMI were also significantly lower in the PM subgroup compared to the IM subgroup or the NM + RM subgroup. However, the 1-year MACCE-free survival rates were only numerically, but not statistically significantly, lower in the PM group in the overall cohort. Other studies from East Asia also indicated increased MACE events in CYP2C19 PM in the setting of AMI but not in the setting of stable angina. Kim et al. reported that the poor metabolizer was significantly associated with a higher risk of MACCE in patients with AMI in Korea (hazard ratio, 2.88; 95% confidence interval, 1.27–6.53; *p* = 0.011). However, this finding was not observed in patients with stable angina (17). From a single center in Japan, in the ACS group, cardiovascular events were higher in carriers of the LOF allele (24.6%) vs. non-carriers (11.1%, *p* < 0.05), but such difference was not observed in the stable angina group (carriers: 14.8%; non-carriers: 7.9%, *p* = 0.078) (18). Another study in China that included only ACS patients showed that the carriage of two CYP2C19 LOF alleles was significantly associated with an increased risk of adverse ischemic events at 1-year follow-up (19). Our results are consistent with these studies in Northeast Asia and demonstrate the impact of CYP2C19 LOF alleles in patients with AMI.

The relationship between CYP2C19 LOF alleles in patients with AMI using clopidogrel and clinical events has been studied in Northeast Asia, whereas studies in Southeast Asia are limited (20, 21). In Taiwan, although the location was near northeast Asian, the island of Southeast Asia (ISEA) ancestry was one of the major genetic ancestries in Taiwan. Lo et al. identified considerable proportions of ISEA ancestry (also carried by many Austronesian speaking populations) in most Taiwanese Han individuals (average 15%, range 0.1–62%) (22). Our result addresses the knowledge gap for patients in Southeast Asia with CYP2C19 LOF alleles using clopidogrel after PCI. The results are consistent with the Singapore cohort (21), which revealed that LOF patients were significantly more likely to experience MACE compared to non-LOF subjects in acute coronary syndrome (8.0 vs. 5.4%, *p* = 0.041). In patients without ACS, another smaller cohort in Malaysia recruited patients who underwent elective PCI (20). The results showed that the presence of the CYP2C19*2 polymorphism was not significantly associated with 1-year MACE after the implantation of DES. Similar results were obtained in our group in the setting without AMI. Our study, the largest cohort in Southeast Asians with nearly 1,000 patients, had approximately four times the number of previous Singapore and Malaysian cohorts. All these studies highlighted the crucial rule of CYP2C19 LOF alleles in Southeast Asians with AMI, but not in patients with stable angina undergoing elective PCI.

In patients with ACS undergoing PCI, current guidelines recommend potent P2Y12 inhibitors, namely ticagrelor or prasugrel, in preference to clopidogrel to reduce ischemic events, including stent thrombosis (3, 23, 24). However, these potent P2Y12 inhibitors lead to more bleeding events. In selected patients with high bleeding risks, some experts suggest de-escalation of potent P2Y12 receptor inhibitors to clopidogrel, either based on clinical judgment, platelet function testing or CYP2C19 genotyping (24). Recently, the TALOS-AMI study found that in Korean patients with AMI after index PCI, a uniform unguided de-escalation strategy switching from ticagrelor to clopidogrel after 1 month significantly reduced the risk of net clinical events up to 12 months, mainly by reducing bleeding events. However, given the high prevalence of the CYP2C19 LOF allele in East Asians, as the well as the high rates of 1-year MACCE in patients carrying the CYP2C19 LOF allele after PCI in AMI, we believe that the unguided de-escalation approach in the TALOS-AMI study should be applied cautiously in East Asians. The results of this study are in agreement with the findings of recent meta-analyses which demonstrated that guided de-escalation of P2Y12 inhibitors reduces bleeding without any trade-off in ischemic events (25–27).

### Study limitations

Our study had several limitations. First, the retrospective study design was inevitably associated with selection bias and other confounding factors. Some critical parameters, such as coronary artery complexity, detailed analysis of antiplatelet duration, and concomitant use of anticoagulant, might not be collected completely in every patient and utilized for outcome analysis. Second, the enrollment of consecutive all-comers after different types of stents, including bare-metal stents, drug-eluting stents and bioresorbable vascular scaffolds, might influence the clinical results. However, this allowed us to investigate the associations of CYP2C19 LOF in patients after PCI taking clopidogrel in a real-world setting. Not surprisingly, the multivariate analysis in our study identified bare-metal stent as independent factors for MACCE both in overall cohort and in the AMI subgroup. However, poor metabolizer was the strongest independent predictor of 1-year MACCE in patients with AMI, which implies the importance of CYP2C19 genotyping in AMI patients. Third, our study focused on the influence of the CYP2C19 genotype. Platelet function tests were not included in the analysis of this study. Fourth, although our study demonstrated that CYP2C19 PM were associated with high 1-year MACCE in AMI, large-scale studies are still warranted to find the pragmatic approach in clinical practice of genotype-guided de-escalation of P2Y12 inhibitors in East Asians.

## Conclusion

In East Asians with AMI, the 1-year MACCE rates, as well as the incidence of stroke, were significantly higher in the CYP2C19 PM subgroup. Poor metabolizer of CYP2C19 was the strongest independent predictor of 1-year MACCE in patients with AMI. These results reinforce the crucial role of CYP2C19 genotyping in East Asian AMI patients receiving clopidogrel therapy.

## Data availability statement

The original contributions presented in this study are included in the article/supplementary material, further inquiries can be directed to the corresponding author.

## Ethics statement

The studies involving human participants were reviewed and approved by the Institutional Review Board of TCVGH (CE20316A). The patients/participants provided their written informed consent to participate in this study.

## Author contributions

Y-WC, Y-JL, W-CC, and Y-MC contributed to the conception and design of the study. Y-WC, Y-JL, W-CC, T-HH, C-HL, and Y-MC contributed to the data collection. C-YH analyzed and interpreted the data. Y-WC drafted the report, which was critically revised for important intellectual content by T-JL, W-LL, and Y-MC. All authors have participated in the work, reviewed and agreed with the content of the article, and approved the final version of the report.
